# Dimethyl fumarate decreases short-term but not long-term inflammation in a focal EAE model of neuroinflammation

**DOI:** 10.1186/s13550-022-00878-y

**Published:** 2022-02-02

**Authors:** S. K. Vainio, A. M. Dickens, M. Matilainen, F. R. López-Picón, R. Aarnio, O. Eskola, O. Solin, D. C. Anthony, J. O. Rinne, L. Airas, M. Haaparanta-Solin

**Affiliations:** 1 Turku PET Centre, Preclinical PET Imaging, Preclinical Imaging Laboratory, University of Turku, Tykistökatu 6 A, 20520 Turku, Finland; 2 MediCity Research Laboratory, University of Turku, Turku, Finland; 3 Department of Chemistry, University of Turku, Turku, Finland; 4 Turku Bioscience, Turku, Finland; 5 Faculty of Science and Engineering, Åbo Akademi University, Turku, Finland; 6 Accelerator Laboratory, Åbo Akademi University, Turku, Finland; 7 Turku PET Centre, Radiopharmaceutical Chemistry Laboratory, University of Turku, Turku, Finland; 8 Department of Pharmacology, University of Oxford, Oxford, UK; 9 Division of Clinical Neurosciences, Turku University Hospital, Turku, Finland; 10 Turku PET Centre, University of Turku, Turku, Finland; 11 Department of Clinical Medicine, University of Turku, Turku, Finland

**Keywords:** Multiple sclerosis, *f*DTH-EAE, Dimethyl fumarate, BG-12, [^18^F]GE-180

## Abstract

**Background:**

Dimethyl fumarate (DMF) is an oral immunomodulatory drug used in the treatment of autoimmune diseases. Here, we sought to study whether the effect of DMF can be detected using positron emission tomography (PET) targeting the 18-kDa translocator protein (TSPO) in the focal delayed-type hypersensitivity rat model of multiple sclerosis (*f*DTH-EAE). The rats were treated orally twice daily from lesion activation (day 0) with either vehicle (tap water with 0.08% Methocel, 200 µL; control group *n* = 4 (3 after week four)) or 15 mg/kg DMF (*n* = 4) in 0.08% aqueous Methocel (200 µL) for 8 weeks. The animals were imaged by PET using the TSPO tracer [^18^F]GE-180 in weeks 0, 1, 2, 4, 8, and 18 following lesion activation, and the non-displaceable binding potential (BP_ND_) was calculated. Immunohistochemical staining for Iba1, CD4, and CD8 was performed in week 18, and in separate cohorts of animals, following 2 or 4 weeks of treatment.

**Results:**

Using the *f*DTH-EAE model, DMF reduced the [^18^F]GE-180 BP_ND_ in the DMF-treated animals compared to control animals after 1 week of treatment (two-tailed unpaired *t* test, *p* = 0.031), but not in weeks 2, 4, 8, or 18 when imaged in vivo by PET. Immunostaining for Iba1 showed that DMF had no effect on the perilesional volume or the core lesion volume after 2 or 4 weeks of treatment, or at 18 weeks. However, the optical density (OD) measurements of CD4^+^ staining showed reduced OD in the lesions of the treated rats.

**Conclusions:**

DMF reduced the microglial activation in the *f*DTH-EAE model after 1 week of treatment, as detected by PET imaging of the TSPO ligand [^18^F]GE-180. However, over an extended time course, reduced microglial activation was not observed using [^18^F]GE-180 or by immunohistochemistry for Iba1^+^ microglia/macrophages. Additionally, DMF did affect the infiltration of CD4^+^ and CD8^+^ T-lymphocytes at the *f*DTH-EAE lesion.

**Supplementary Information:**

The online version contains supplementary material available at 10.1186/s13550-022-00878-y.

## Highlights

In a focal rat DTH-EAE model of neuroinflammation, dimethyl fumarate decreases the uptake of TSPO PET tracer [18F]GE-180 in the short term.

Long-term [^18^F]GE-180 follow-up did not indicate a treatment effect.

Decreased neuroinflammation, CD4^+^ T cell infiltration, and CD8^+^ T cell infiltration were detected using immunohistochemistry.

**Supplementary Information:**

The online version contains supplementary material available at 10.1186/s13550-022-00878-y.

## Background

Dimethyl fumarate (DMF, BG-12) is an oral first-line therapy for multiple sclerosis (MS) [1, 2] that received FDA approval in 2013, but the precise mode of action in MS remains unclear. In human studies, DMF reduces relapse rate and radiological signs of disease activity [3]. Its primary metabolite, monomethyl fumarate, also appears to contribute to the therapeutic effect, which may be both immunomodulatory and neuroprotective [4]. It has been shown that DMF activates the nuclear factor erythroid 2-related factor 2 (Nrf2), and therefore, it can be argued to have antioxidant effects. However, the impact of DMF on microglial and lymphocyte function in vivo is less well studied [5, 6]. DMF has been shown to reduce T cell and macrophage infiltration into the spinal cord of a mouse EAE model [7] and across human brain endothelial cells in vitro [8].

Within active MS lesions, the presence of ‘activated’ microglia is a characteristic feature, and their presence contributes to demyelination, axonal injury, and neuronal loss. Activated microglia in MS lesions can be visualised using PET- or SPECT-based molecular imaging with ligands that bind to the mitochondrial 18-kDa translocator protein (TSPO).

In the central nervous system, microglia acquire dysfunctional phenotypes when preserving brain homeostasis [9]. DMF has been shown to reduce the T cell migration potential in MS patients [10], whereas the effect of DMF on microglial functions is unclear [11]. Thus, we wanted to study the effect of oral DMF on microglial activation using positron emission tomography (PET) and immunohistochemistry (IHC). Experimental autoimmune encephalomyelitis (EAE) is widely used in animal studies of MS. We decided to use a focal delayed-type hypersensitivity (*f*DTH)-EAE rat model of MS [12] because of its focal nature and usability in imaging studies [13]. Disseminated EAE models have previously been treated with DMF, with preventive use reducing disease activity [14]. Here, the treatment was started on the day of lesion activation.

In this study, we investigated whether a treatment effect of DMF can be detected by TSPO PET imaging using the tracer [^18^F]GE-180 and by IHC. We sought to determine whether DMF would reduce microglial activation and the number of T lymphocytes in the lesions of the *f*DTH-EAE rat model of MS. In addition, we wanted to determine whether a rebound effect occurs after withdrawing the treatment, as a report of rebound after discontinuation of DMF has been described [15].

## Methods

### Animals

Animal experiments were carried out according to ARRIVE guidelines 2.0 [16]; the United Kingdom Animals (Scientific Procedures) Act, 1986; and EU Directive 2010/63/EU for animal experiments. The experiment received ethics approval from the Finnish National Animal Experiment Board (ESAVI/6360/04.10.03/2011). Male Lewis rats (*n* = 24) were obtained from Charles River Laboratories (Sulzfeld, Germany) and acclimatised for 7 days before the start of the experiments. Animals were housed in accordance with the Treaty of Amsterdam protocol for animal welfare, in pairs in individually ventilated cages with a consistent temperature of 21 (1.2)°C and consistent humidity of 55 (5)% with a 12-h light/dark cycle. Food (CRM(E) Expanded, Special Diet Services, UK) and tap water were provided ad libitum. Values are indicated as mean (SD).

**Fig. 1:**
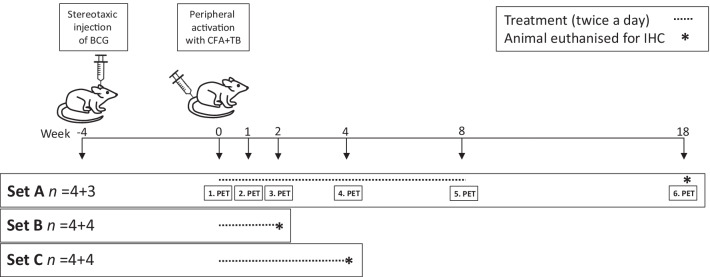
Study timeline. All animals (*n* = 24) were operated in week—4 and heat-killed bacillus Calmette–Guérin (BCG, 10^5^ organisms in 2 μL of phosphate-buffered saline) was injected stereotaxically in the right striatum over 10 min. At week 0, the animals were intradermally injected with heat-killed mycobacterium tuberculosis (TB; 1,5 mg) in complete Freund’s adjuvant (CFA) to activate the brain lesion peripherally. Dosing of dimethyl fumarate (DMF; 15 mg/kg) or vehicle (tap water with 0.08% Methocel) using oral gavage was started on day 0 in week 0. Set A animals had a baseline PET study on the same day. Set A animals were imaged during the treatment regimen in weeks 1, 2, 4, and 8, as well as 10 weeks after the treatment finished (i.e. week 18). One Set A animal from the control group died after imaging in week 4. Set B animals were euthanised for immunohistochemistry (IHC) in week 2 and set C animals were euthanised for IHC in week 4

**Fig. 2:**
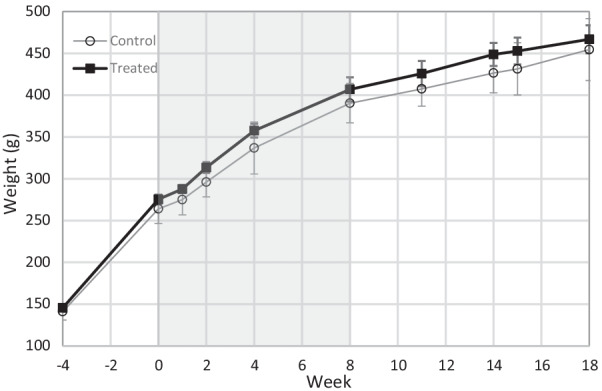
Weight gain of the PET-imaged animals in Set A (*n* = 8). There were no significant differences between the control (*n* = 4 or 3 after week 4) and treated (*n* = 4) animals at the individual time points using multivariate analysis. The results are presented as mean (SD). The DMF treatment period is indicated as grey background

The animals were randomly assigned to the treatment groups (Fig. 1). The *f*DTH model of MS was induced in the same manner as described previously [13]. Briefly, to induce the *f*DTH lesion, heat-killed bacillus Calmette–Guérin (BCG, 10^5^ organisms in 2 μL of phosphate-buffered saline) was injected in the right striatum (RC + 1.0 mm, ML + 3.0 mm, DV-4.0 mm from bregma). The injection was performed in four 0.5 µL volumes by using a Hamilton syringe (Sigma-Aldrich) within the dorsal ventral depth − 4.0–2.5 mm over 10 min to avoid back-flow of the thick BCG. Four weeks after the intracranial injection of BCG (i.e. at week 0), the animals were peripherally sensitised by intradermal injection of heat-killed mycobacterium tuberculosis (TB, 1.5 mg, Difco Laboratories, Detroit, MI, USA) in an emulsion of complete Freund’s adjuvant (CFA, 50:50 v/v FA/saline, 100 μL, Sigma Aldrich, Saint Louis, MO, USA) into the flank of the rat, which initiates an *f*DTH lesion at the site of the intracranial injection of heat-killed BCG. This lesion activation step, at 4 weeks after the intracranial microinjection of BCG, is recorded as experimental day 0. The study animals were divided into three sets of eight rats each (*n* = 4 treated and *n* = 4 control): Set A was imaged by PET and euthanised for IHC after the last PET study, Set B was used for IHC after 2 weeks of treatment, and Set C used for IHC after 4 weeks of treatment. These time points were chosen to correspond with the time points of PET imaging in Set A. The time points for PET imaging were chosen for baseline imaging, acute phase of inflammation (at 1–2 weeks) and chronic phase (at 4–8 weeks) and to evaluate the potential rebound effect after halting the treatment for 10 weeks (at 18 weeks). Weight gain was measured throughout the imaging period (Fig. 2).

Starting on day 0, the rats were treated twice daily *per os* with 15 mg/kg of DMF (Sigma‐Aldrich Chemie GmbH, Steinheim, Germany) diluted in 0.08% aqueous Methocel (200 µL, Sigma‐Aldrich Chemie GmbH, Steinheim, Germany), or vehicle (tap water with 0.08% Methocel) for controls, using an oral gavage. Dosing of DMF was based on previous reports [7, 17]. Methocel was used because of the poor solubility of DMF in water [7]. To aid the dissolution of DMF, the solution was sonicated for 15 min.

### Radiochemistry

[^18^F]GE-180 ((S)-*N*,*N*-diethyl-9-(2-[^18^F]fluoroethyl)-5-methoxy-2,3,4,9-tetrahydro-1*H-*carbazole-4-carboxamide) was synthesised at the Radiopharmaceutical Chemistry Laboratory of Turku PET Centre as described previously [18]. The molar activity of [^18^F]GE-180 was 1.6(0.2) TBq/μmol at the end of syntheses (*n* = 6). The radiochemical purity was ≥ 97.8%.

### PET

Animal PET imaging was performed using an Inveon Siemens multimodality PET/computed tomography (CT) scanner (Siemens Medical Solutions USA, Knoxville, TN) designed for small laboratory animals. The rats were anaesthetised with isoflurane/air (4% isoflurane with 700 mL/min air for induction and 2–3.0% isoflurane with 400–500 mL/min air for maintenance on the table, until the animal was placed in the PET scanner) 20 min before the [^18^F]GE-180 injection. During the PET scan, the rats were under anaesthesia in 2–2.5% isoflurane/700 mL oxygen. Body temperature was maintained during imaging using a heating pad on which the rats slept. Two animals were imaged at once. Following transmission scans for attenuation correction using the CT modality, static PET images (5 × 300 s frames) were obtained 25–50 min after the injection of [^18^F]GE-180 with an energy window of 350–650 keV. The injected activity for DMF-treated animals was 32.23(1.27) MBq and the injected mass 28.4(13.4) ng/kg. The injected activity for the control animals was 32.56(1.18) MBq and the injected mass 29.2(12.9) ng/kg.

The PET data were reconstructed using the ordered-subsets expectation maximisation algorithm in three dimensions (OSEM3D) twice and MAP iterative reconstruction protocols 18 times in the Inveon™ acquisition software (Siemens Medical Solutions, USA).

Images were analysed using PMOD analysis software (v3.4 PMOD Technologies Ltd., Zürich, Switzerland). Images were divided and aligned to the Schiffer MR template that is inbuilt into the software by first aligning the PET to the CT space, after which the CT was aligned to the MR space. Alignment of the PET image to the MR space was achieved by combining the transformations, applying manual supervision and motion correction whenever needed. The images were summed to standardised uptake value (SUV) maps (25–50 min).

The volume of interest (VOI) was drawn with the Automatic Isocontour Detection tool for each animal individually by choosing the stage of the lesion at its largest (varied between individuals from week 1 to week 2) and applying this VOI to all other time points within one individual. In addition, a contralateral, spherical VOI was drawn on the contralateral striatum. The non-displaceable binding potential (BP_ND_) was calculated as: (ipsilateral uptake − contralateral uptake) / contralateral uptake.

### Immunohistochemistry

Animals were perfuse-fixed with periodate–lysine–paraformaldehyde fixative with 0.1 vol% glutaraldehyde and cryoprotected with sucrose. The method has been described in more detail previously [13, 19]. Staining was performed in a semiautomatic LabVision autostainer (Thermo-Fisher Scientific). Sections were pre-heated in citrate buffer (pH 6, Genmed), blocked with hydrogen peroxide and pre-protein block (Draco antibody diluent; WellMed), and incubated with either Iba1-Ab (Wako, 1:2000 dilution, RT), anti-CD4 (Abcam, ab33775, dilution 1:50), or anti-CD8 (Abcam, ab33786, dilution 1:200) for 60 min at room temperature (RT). For the anti-Iba1 staining, the Orion 1 step detection system (Goat anti-rabbit HRP; WellMed) was used as a secondary antibody (Ab) for 30 min at RT. For anti-CD4 and anti-CD8 staining, the Bright vision 1 step detection system (Goat anti-mouse HRP) was applied. The sections were stained with DAB (Taurus; WellMed) and Mayer’s haematoxylin.

Anti-Iba1 staining was performed to detect activated microglia within and around the lesion core. Ten-micrometre-thick brain sections were obtained with a gap of a 100 µm through the *f*DTH lesion area of 750 μm from the lesion centre on either side. The Iba1-immunopositive area of the perilesional area or the hypercellular core (depicted in Fig. 4d) was assessed using CaseViewer 2.1 software (3DHISTECH Ltd., Budapest, Hungary). The semi-quantitative volume calculation was performed by drawing the area of activated microglia for each brain section and extrapolating the area in between using the trapezoidal rule. The perilesional volume (Fig. 4a) and the lesion core volume (Fig. 4b) were calculated for both the control and the DMF-treated animals.

To detect T lymphocytes, brain sections (*n* = 3) adjacent to the lesion core with anti-Iba1 staining were selected. Because individual CD4^+^ and CD8^+^ T cells (Fig. 5) could not be calculated, quantitation was performed by measuring the optical density (OD) of a region of interest (ROI) at the lesion core, in the perilesional area, and at the contralateral site corresponding to the lesion core on the ipsilateral side of the coronal brain section. The image was deconvolved to an 8-bit image, which corresponds to the DAB staining, using Fiji software (ImageJ v1.52p). It is, on this image, that the ROIs were drawn. OD_ROI_ was calculated using the formula log(maximum intensity/mean intensity_ROI_), where maximum intensity is 255 that corresponds to white in an 8-bit image, and mean intensity_ROI_ is the mean intensity from the lesional or perilesional area obtained from three brain sections within one individual [20, 21]. The function is logarithmic since the signal from a microscopy image is nonlinear. The final OD count was obtained by reducing the contralateral OD_ROI_ from the lesion or perilesional OD_ROI_, i.e. OD = OD_ROIlesion or perilesion_ − OD_ROIcontra_.

### Statistical analyses

The statistical analysis for the weight gain in Set A animals (Fig. 2) at all studied time points (weeks—4, 0, 1, 2, 4, 8, 11, 14, 15, and 18) was performed using a linear mixed model with a compound symmetry covariance structure. The model included a time factor, a group factor, and their interaction. The interaction term was used to assess whether the change over time was significantly different in the DMF-treated group compared to the control group. In case the interaction term was significant, the differences in changes at all time points were checked in a post hoc analysis. The logarithm of the response was used instead of the original values for the model to fulfil the normality assumption. With the original values, the assumptions were violated due to non-normal distribution of the values.

Statistical analysis of the change in BP_ND_ compared to the baseline was analysed using a two-tailed unpaired Student’s t-test for each time point separately (GraphPad Prism 9, GraphPad Software, San Diego, CA, USA), and additionally, in the same manner as the weight gain analysis, by using a linear mixed model with a compound symmetry covariance structure. However, the logarithm of the response was used instead of the original values for the model to fulfil the normality assumption.

Anti-CD4 and anti-CD8 staining data, with OD as the response, were analysed separately using linear mixed models with compound symmetry covariance structures. The models included an OD_ROI_ factor (lesion or perilesional area), a group factor (DMF-treated or control), week as a group factor with a separate set of subjects for weeks 2 and 4, and the interaction term between OD_ROI_ and group, with possible post hoc analysis in case of significance. The square root of the response was used instead of the original values for the model to fulfil the normality assumption in both models.

In post hoc analyses, *p*-values were adjusted using the Tukey–Kramer method. The normality assumption was checked using the studentised residuals. The linear mixed model analyses were performed using SAS version 9.4 for Windows (SAS Institute Inc., Cary, NC, USA). All statistical tests were two-sided with the significance level set at 0.05. Values for animal weight data, injected radiochemical masses, BP_ND_ and OD are reported as means (SDs).

## Results

### Animals

All animals (*n* = 24) recovered from the *f*DTH model-induction procedures and showed no clinical manifestation of neuroinflammation, when observed daily for signs of neurological symptoms. During the week 4 PET study, one control animal from Set A died in the PET scanner, owing possibly to an overdose of anaesthesia, but we retained the early time points, i.e. week 0, 1, and 2 results, in the PET analysis. A focal lesion was detected by IHC in all but one animal.

Body weight increased significantly (Fig. 2) in both the control and DMF-treated groups (*p* < 0.001) throughout the study period and between almost all time points. We found no significant differences in weight change over time between the DMF-treated group and the control group (*p* = 0.356) at each time point. In addition, weight did not differ between the groups (*p* = 0.158).

### PET

**Fig. 3:**
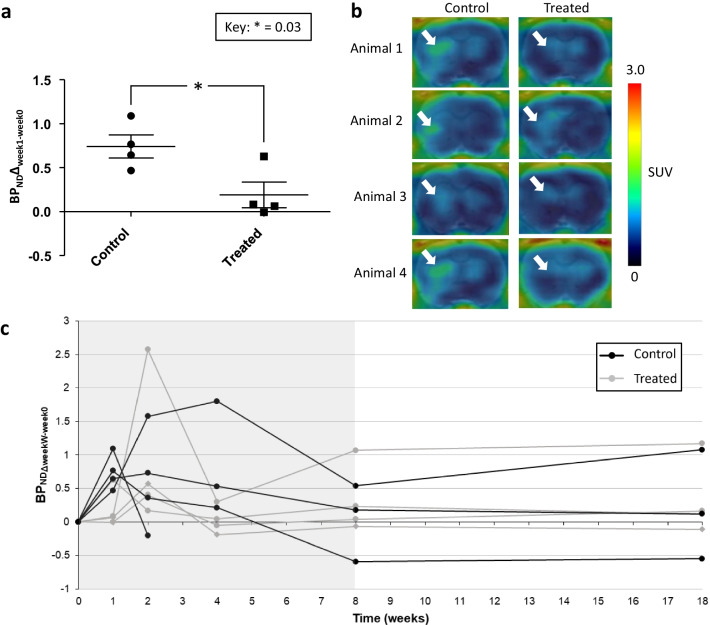
Results from in vivo PET imaging. **a** Results from week 1 indicated a treatment effect of dimethyl fumarate (DMF). The binding potential (BP_ND_Δ_week1-week0_) was calculated by reducing the BP_ND_ of week 1 from the baseline image of an individual using a constant VOI for each animal for each time point. Statistics were calculated using the Student’s t-test. The results are presented as mean (SD). Values in this figure represent the changes compared to baseline and not the original values to aid comparability of the BP_ND_ between different individuals. However, the original values were applied in the statistical analyses. **b** Representative week 1 images. **c** Longitudinal in vivo imaging showed no differences between the control and DMF-treated animals at any time point when analysed using a linear mixed model. Binding potential was calculated by first calculating: (BP_ND_) (ipsilateral uptake − contralateral uptake)/ contralateral uptake. After this, BP_ND_Δ_weekW-week0_ was calculated by reducing the BP_ND_ of the specific week (W = 1/2/4/8/18) from the baseline image of an individual using a constant VOI for each animal through the different time points. The treatment period for Set A animals is indicated as grey background in the graph

The [^18^F]GE-180 images were analysed by comparing the BP_ND_ of the specific week (W = 1/2/4/8/18) to the baseline BP_ND_ at week 0 (BP_ND_Δ_weekW-week0_). The analysis revealed that the BP_ND_ of [^18^F]GE-180 at week 1 (BP_ND_Δ_week1-week0_) was reduced in the DMF-treated group compared to the control group in the *f*DTH model (*p* = 0.031; Fig. 3a, b, Additional file 1: Fig. 3). However, we found no significant differences in the changes in BP_ND_Δ_weekW-week0_ over time between the DMF-treated group and control group (*p* = 0.142) in weeks 1, 2, 4, and 8 (Fig. 3c). Furthermore, we did not detect a rebound effect in week 18 after halting the DMF treatment in week 8 (Fig. 3c). The values in Fig. 3a, c represent the changes compared to baseline and not the original values to aid comparability of the BP_ND_ between different individuals. However, the original values were applied in the statistical analyses.

The BP_ND_ values did not differ between the groups overall (*p* = 0.614). However, we did find significant changes in BP_ND_Δ_weekW-week0_ over time among all individuals (*p* = 0.024), especially between the baseline imaging and 1-week follow-up (*p* = 0.018), as well as baseline and 2-week follow-up *p* = 0.030) when looking at unadjusted *p*-values (Fig. 3c). A similar significance was also detected between week 2 and week 4 follow-up (*p* = 0.035), week 2 and week 8 follow-up (*p* = 0.014), as well as week 2 and week 18 follow-up (*p* = 0.032) when looking at unadjusted *p*-values.

### Immunohistochemistry

**Fig. 4:**
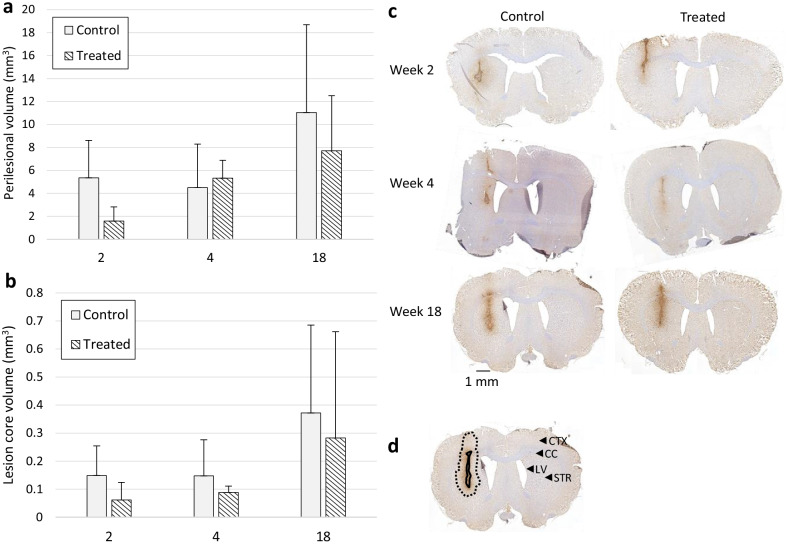
Immunohistochemical staining against Iba1 in weeks 2, 4, and 18. Nuclei are counterstained with haematoxylin. **a**, **b** The volume was determined semi-quantitatively by calculating the area of activated microglia at 100-µm intervals and extrapolating the area in between using the trapezoidal rule. **a** The perilesional volume (mm^3^) of the lesion and **b** the lesion core volume were calculated as an average for both the control and treated animals. No differences were detected at any time points between the treated and control animals. The results are presented as mean (SD). **c** Representative images of the control and treated animals in weeks 2, 4, and 18. Scale bar = 1 mm. **d** A representative image of the lesion core (solid black line) and perilesional area (dotted black line) delineation of the week 18 control animal. The lesion core indicates the infiltrative core area of the lesion with high Iba1 staining. The perilesional area is a subjective estimate of the area that has increased diffuse microglial activation higher from the contralateral staining but less prominent as the lesion core. In addition, the prominent brain regions visible on the coronal sections have been indicated on the contralateral side by using arrow-heads. *CTX* cortex, *LV* lateral ventricle, *CC* corpus callosum, *STR* striatum

The semi-quantitative analysis performed against Iba1, indicating microglial activation and infiltrated macrophages (Fig. 4c), showed no differences in lesion size between the control and DMF-treated animals when calculating the perilesional volume (Fig. 4a) or the lesion core volume (Fig. 4b).

**Fig. 5:**
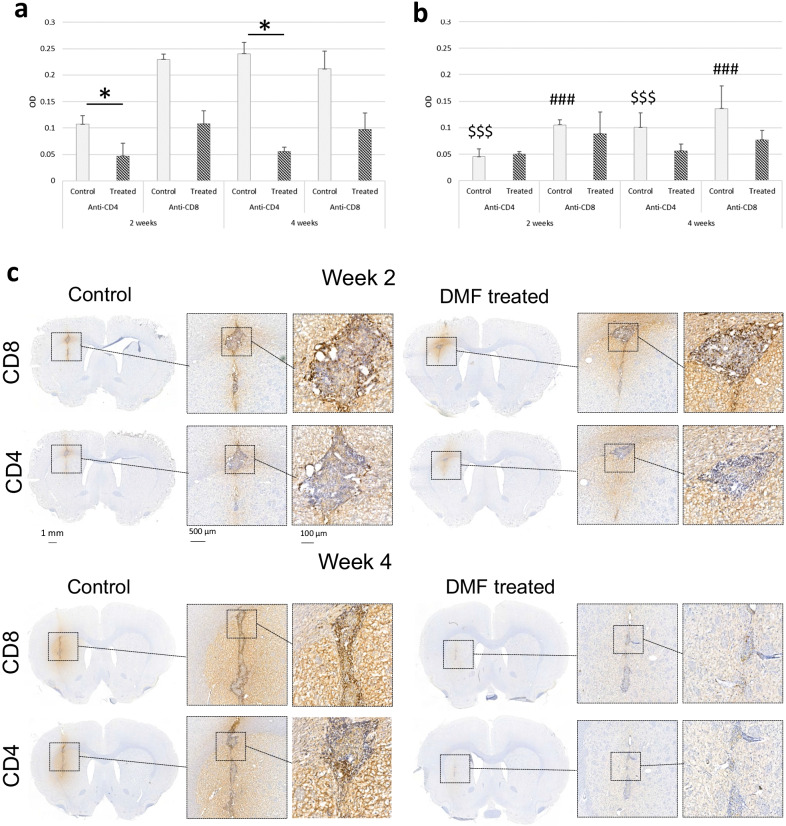
Immunohistochemical staining of T lymphocytes. Staining was performed against CD4 and CD8 for control and DMF-treated animals in Set B (*n* = 8) and Set C (*n* = 8) 2 and 4 weeks after treatment with vehicle or DMF. Optical density (OD) was calculated by subtracting the contralateral OD_ROI_ from the lesion/perilesional OD_ROI_. **a** The OD of CD4^+^ and CD8^+^ T lymphocytes at the lesion core. The asterisk (*) denotes significant differences between DMF-treated and control animals. **b** The corresponding OD in the perilesional area. In both the anti-CD4 and anti-CD8 staining, the control group had significantly higher OD values at the lesion core (**a**) than in the perilesional area (**b**) (*p* < 0.001). These differences were not found in the treated group (anti-CD4: *p* = 1; anti-CD8 *p* = 0.2) in post hoc analysis. Controls had significantly higher CD4^+^ OD values at the lesion core (**a**) compared to the DMF-treated group (*p* = 0.041), but this was not detected with CD8. The dollar sign ($) indicates significant differences between the CD4^+^ OD of the control animals at the focal lesion core (**a**) compared to the perilesional area (**b**) (*p* < 0.001). The number sign (#) indicates significant differences between the CD8^+^ OD of the control animals at the lesion core (**a**) compared to the perilesional area (**b**) (*p* < 0.001). The results are presented as mean (SD). **c** Representative immunohistochemical staining against CD4^+^ and CD8^+^ T lymphocytes in the lesion core in the control and DMF-treated animals. Nuclei are counterstained with haematoxylin. Scale bar = 500 µm, or 100 µm in the enlarged image

To detect lymphocyte infiltration, staining was performed against CD4^+^ and CD8^+^ T cells (Fig. 5). Anti-CD4^+^ and anti-CD8^+^ T lymphocytes were present at the lesion core in both control and DMF-treated animals in week 2 (i.e. Set B) and week 4 (i.e. Set C; Fig. 5c). For anti-CD4 staining, the group and OD_ROI_ interaction were significant, i.e. the effect of OD_ROI_ differs significantly in different groups (*p* < 0.001). The control animals had significantly higher OD values at the lesion core (Fig. 5a) compared to the perilesional area (Fig. 5b; *p* < 0.001), but such differences were not found in the DMF-treated group (*p* = 1) in the post hoc analysis. Furthermore, the control animals had significantly higher OD values at the lesion core (Fig. 5a) compared to the DMF-treated group (*p* = 0.041). We found no differences between weeks 2 and 4 (*p* = 0.3), and no differences in OD were detected in the perilesional area (Fig. 5b).

For anti-CD8 staining, the group and OD_ROI_ interaction was significant, i.e. the effect of OD_ROI_ differs significantly in different groups (*p* = 0.012). The control animals had significantly higher OD values at the focal lesion core (Fig. 5a) compared to the perilesional area (Fig. 5b; *p* < 0.001), whereas such differences were not found in the DMF-treated group (*p* = 0.2) in post hoc analysis. The difference in lesion OD values between the control and DMF-treated groups was not significant (*p* = 0.120). In addition, there was no difference between weeks 2 and 4 (*p* = 0.9), and no differences in OD in the perilesional area (Fig. 5b).

## Discussion

In this study, the effect of DMF on a *f*DTH-EAE rat model was studied using the TSPO PET tracer [^18^F]GE-180 and IHC. [^18^F]GE-180 has been shown to detect neuroinflammation in animal models [22] and can be used as a marker of neuropathological changes in animal models of MS [23]. By using [^18^F]GE-180, we were able to detect a reduced uptake in the *f*DTH-EAE rats after 1 week of twice daily treatment with DMF *per os* compared to the vehicle-treated control group. However, no therapeutic effect was seen after the first week of treatment with DMF compared to the groups by PET using [^18^F]GE-180. Clearly, at the later time points, more power would have been needed to confirm the result. Student's t-test was performed initially to see whether there is a difference between the changes from baseline to a specific week. In the mixed model, all the differences between all the time points were also considered, and thus, important changes from baseline that we are actually interested in may have lost their significance after multiple comparison corrections. In addition, [^18^F]GE-180 may not be specific enough to detect the cellular changes in inflammation in this model, which include infiltration of T cells, monocyte recruitment, axonal and myelin damage, upregulation of matrix metalloproteinases, and microglial activation. DMF is affecting mostly the lymphocyte composition; thus, the changes in TSPO expression may be moderate.

As a limitation of the study, we can mention high variability between subjects when studying TSPO expression. There is individual variation in the animal’s response to lesion induction, despite the efforts to standardise the surgical procedure. This has been detected in our previous studies as well [24]. Furthermore, due to the thickness of BCG, there is always backflow of the substance through the injection track. If the BCG reaches the cortical surface of the brain, spontaneous lesions may occur and cause variation in the lesions [25].

Our analyses also showed that radiometabolites were present in the rat brain (Additional file 1: Supplemental data), which has the consequence that the radioactivity signal is derived, not only from the tracer, but also from its radioactive metabolites. Previous studies have reported the presence of radiometabolites in the rat brain [26]. Analysis of the binding of plasma radioactive components to plasma proteins (Additional file 1: Supplemental data) indicated that the radioactive metabolites would be less bound to plasma proteins than the tracer [^18^F]GE-180. Only the free fractions of the parent and radiometabolites are free to cross the BBB [27–29]; thus, even the less lipophilic radiometabolites may transport across the BBB due to their elevated free fraction.

In clinical studies, [^18^F]GE-180 has been shown to detect active lesions in relapsing–remitting MS (RRMS) due to tracer penetration through the disturbed BBB [30], though the tracer distribution volume is low [31], and its BBB penetration through an intact BBB in humans is poor [32]. Importantly, TSPO expression in rodents reflects the activation phenotype of microglia after pro-inflammatory activation and the infiltration of macrophages, whereas in humans it may reflect microglia and macrophage density [33].

Although rebound after discontinuation of DMF therapy has been reported previously [15], we observed no changes in [^18^F]GE-180 uptake 10 weeks after the discontinuation of treatment, indicating no rebound effect. However, we cannot rule out a rebound effect prior to study week 18, which was the time point used in this study for practical reasons.

Clinical use of oral DMF in RRMS has demonstrated the efficacy and ease of use with tolerable adverse effects, which is why it is a first-line treatment [34, 35], even though the mechanism of action of the drug is not fully understood [36]. Here, we were not able to detect a reduced lesion size in the *f*DTH-EAE model using anti-Iba1 staining against activated microglia/macrophages after 2 or 4 weeks of DMF treatment.

DMF has been shown to reduce both the CD4^+^ and CD8^+^ lymphocyte counts in patients with RRMS [37]. Therefore, IHC was performed against CD4^+^ and CD8^+^ T lymphocytes. Interestingly, both CD4^+^ and CD8^+^ stainings were detected not only at the lesion core at the site of lymphocyte infiltration, but also as a halo in the perilesional area. Therefore, we calculated the OD for both the lesion core and the perilesional area. The control group had significantly higher OD values in the lesion core (Additional file 1: Fig. 2), which may be due to the presence of lymphocytes with high expression of CD4 and CD8 compared to the microglia in the perilesional area. In addition, DMF treatment reduced the staining of CD4 at the lesion core, which indicates that DMF reduced the infiltration of T cells at the lesion core but did not affect the perilesional staining.

The halo of CD4^+^ and CD8^+^ cells corresponds to the anti-Iba1-staining of microglia, possibly indicating the expression of CD4 and CD8 in the activated microglia in addition to the T cells. Microglia were previously reported to express CD4 [38, 39]. In addition, increased CD8 signalling has been shown to be associated with microglial activation and macrophages in post-stroke brain damage [40]. While expressing both CD4 and CD8, the neuroinflammatory damage in the *f*DTH lesion seems to promote polarisation of the microglia into the pro-inflammatory subtype. The IHC data were collected from samples 2 and 4 weeks after treatment, but unfortunately not at week 1, which would correspond with the PET data.

Studies of the pharmacokinetic properties of DMF have reported that the pharmacokinetics are quick, but dosing with DMF twice or thrice a day does not alter the therapeutic outcome in humans [41]. When this experiment was performed, preventive dosing with 15 mg/kg twice a day in EAE mice had been reported to affect the clinical symptoms of EAE [7]. Thus, we started dosing with DMF on day 0, the day of peripheral activation with CFA and TB, to aim for the acute period before lesion activation in the CNS. However, a study by Lin and colleagues (2016) using DMF doses of 25 mg/kg and 50 mg/kg showed dose-dependence of DMF efficacy when treating EAE [42] and thus recommended a higher dose of medication.

DMF has been reported to potentially have gastrointestinal effects [43], which are possibly mediated by hydroxy-carboxylic acid receptor 2 (HCA_2_) [44] and manifest as less weight gain. We followed the weight gain of the rats (Fig. 2) and did not observe this effect with the *f*DTH-EAE Lewis rats treated with DMF during weeks 0–8. The treated and control rats did not differ in weight at any of the measurement time points during the 18-week follow-up. Therefore, we do not expect any gastrointestinal effects in rats during DMF treatment.

## Conclusions

DMF reduces the uptake of TSPO PET tracer [^18^F]GE-180 in the *f*DTH model after 1 week of treatment, but *f*DTH lesion formation is not affected by the DMF treatment in the long term when imaged with [^18^F]GE-180 and IHC staining against Iba1. However, a reduction of infiltrating CD4^+^ T lymphocytes can be detected at the lesion core in the DMF-treated group. In addition, the control group had higher CD4^+^ and CD8^+^ OD in the lesion core when compared to the perilesional area.

## Supplementary Information

**Additional file 1**. Supplemental data.

## Data Availability

The datasets of this article are available upon request from the corresponding author.

## Abbreviations

[^18^F]GE-180: (*S*)-*N*,*N*-Diethyl-9-(2-[^18^F]fluoroethyl)-5-methoxy-2,3,4,9-tetrahydro-1*H*-carbazole-4-carboxamide
Ab: Antibody
ANOVA: Analysis of variance
BP_ND_: Non-displaceable binding potential
BBB: Blood–brain barrier
BCG: Bacillus Calmette–Guérin
CD4: Cluster of differentiation 4
CD8: Cluster of differentiation 8
DMF: Dimethyl fumarate
*f*DTH-EAE: Focal delayed-type hypersensitivity experimental autoimmune encephalomyelitis
IHC: Immunohistochemistry
MS: Multiple sclerosis
Nrf2: Nuclear factor erythroid 2-related factor 2
OD: Optical density
OSEM3D: Ordered-subsets expectation maximisation algorithm in three dimensions
PET: Positron emission tomography
ROI: Region of interest
RRMS: Relapsing–remitting multiple sclerosis
SUV: Standardised uptake value
TSPO: 18-KDa Translocator protein
VOI: Volume of interest
