# Supplementary material for: Dimethyl fumarate decreases short-term but not long-term inflammation in a focal EAE model of neuroinflammation
Source: EJNMMI Res. 2022 Feb 2;12:6. doi: 10.1186/s13550-022-00878-y (PMC8811048; doi:10.1186/s13550-022-00878-y)
Supplement: Supplementary file 1 — Additional file 1. Supplemental data. [file 13550_2022_878_MOESM1_ESM.docx]

**SUPPLEMENTAL DATA**

## METHODS

### Parent and radiometabolite analysis for [^18^F]GE-180

Radiometabolites of [^18^F]GE-180 were analysed from rat plasma and brain tissue at 5, 15, 30, and 60 min (Sprague Dawley rats, n = 1 per time point) in order to understand the parent fraction characteristics of the [^18^F]GE-180 radiopharmaceutical. A thin-layer chromatography method coupled with autoradiography digitation (radioTLC) was developed to measure the minor radiometabolites.

Blood samples were collected via cardiac puncture under deep isoflurane anaesthesia into heparinized Microtainer tubes (BD, Franklin Lakes, NJ, USA) and centrifuged (4 min, 3030 g). The separated plasma was mixed with methanol:plasma (3:2, vol/vol), vortexed, and centrifuged to precipitate proteins.

The brain was excised and homogenized in methanol:water (9:1, vol/vol) using a glass homogenizer. The homogenate was centrifuged (4 min, 3030 g) to obtain clear, protein-free homogenate.

A radioactive standard was prepared to determine the correct retardation factor (R_f_-factor) to distinguish the parent compound from the radiometabolites.

The obtained plasma supernatant, brain homogenate supernatant, and radioactive standard were applied with a pipette (10 µL) on a high-performance TLC plate (HPTLC, silica gel 60 RP-18, art no. 1.05914.0001, Merck KGaA, Darmstadt, Germany). The HPTLC plate was dried and eluted with methanol:water (9:1) in a Twin Trough Chamber (Camag, Muttenz, Switzerland). The migration distance was 4 cm. The dried plate was exposed to an autoradiography plate (BAS-TR2025, Fuji, Tokyo, Japan) for four hours and scanned by phosphorimager (BAS-5000, Fuji, Tokyo, Japan).

The parent fraction was calculated as the percentage of unchanged [^18^F]GE-180 over the total radioactivity of the sample.

### Plasma protein binding of [^18^F]GE-180 and radiometabolites

Ultrafiltration was used to characterize the plasma free fraction of the radioactivity. A plasma sample from each animal was applied to the TLC plate (silica gel 60, art no. 1.05554.0001, Merck KGaA, Darmstadt, Germany). The remaining plasma was ultrafiltered using a 30-kDa cut-off ultrafilter (20 min, 1300 g) with regenerated cellulose membrane (Centrifree® Ultrafiltration Centrifugal Filters, Merck KGaA, Darmstadt, Germany), and a sample of the filtrate (5 µL) from each animal was placed on the same TLC plate as the plasma samples. The TLC plate was dried and scanned identically as for the radiometabolite analysis.

The radioactivity concentration of the filtrate (protein-free analyte) was divided by the radioactivity concentration of the original plasma (protein free + bound analytes) to gain the free fraction of the radioactivity in plasma.

## RESULTS AND DISCUSSION

### Parent and radiometabolite analysis for [^18^F]GE-180

The radioTLC method showed up to five clearly distinguishable radiometabolites in plasma, whereas previous studies with HPLC showed up to three radiometabolites [1] (Fig. 1a). The parent fraction in rat plasma followed a hill function as expected. The unmetabolized fraction in plasma was 81, 55, 31, and 23% at 5, 15, 30, and 60 minutes, respectively. The results were comparable to an earlier study with rat plasma [1]. The metabolism in rat plasma was faster than the metabolism found in human plasma [2].

The parent fraction in brain was 66–77% at all time points (Fig. 1b), which was slightly lower than in the study performed by Boutin *et al.* in 2014 [1]. In addition, Liu *et al.* [3] reported slightly higher fraction results analysed by HPLC in mouse brain than what we found. Plasma results from Liu *et al.* [3] were not reported due the low radioactivity in their plasma samples [3].

**a**


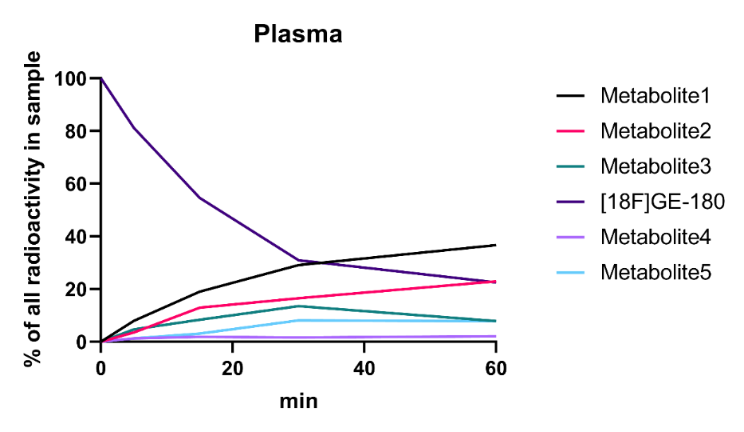

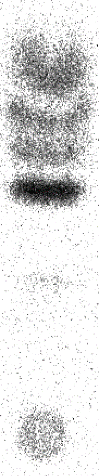


**b**


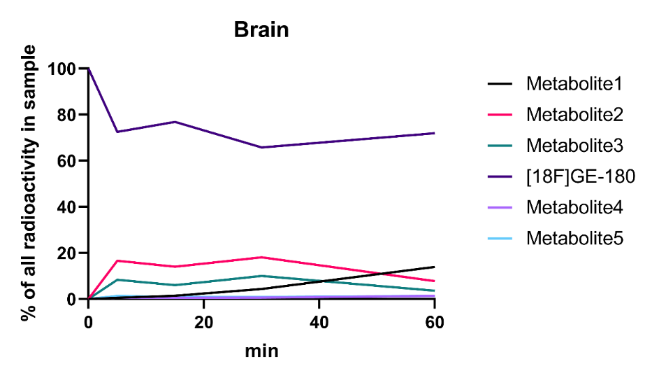

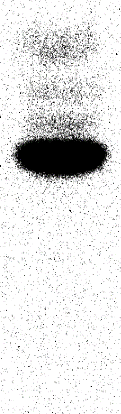


**Suppl. Figure 1.** *In vivo* metabolism of [^18^F]GE-180 in plasma and brain tissue from Sprague Dawley rats (n = 1/timepoint). Time–%-of-radioactivity curves obtained from thin-layer chromatograms of plasma (**a**) and brain (**b**) tissue samples taken at 5, 15, 30, and 60 min after injection of [^18^F]GE-180, which was rapidly metabolised into radiolabelled metabolites. Thus, the fractions of parent tracer and radiolabelled metabolites changed with time. Representative autoradiographs (right) show the non-metabolised and metabolised tracer fractions at 30 min after tracer injection.

### Plasma protein binding of [^18^F]GE-180 and radiometabolites

Using the rapid ultrafiltration method for *ex vivo* plasma samples allows determination of the fraction of radioactivity in plasma that is not bound to proteins. The free fraction was 0.09, 0.31, 0.49, and 0.72 at 5, 15, 30, and 60 min after [^18^F]GE-180 injection, respectively. The increasing free fraction can be explained by the increasing fraction of radiometabolites present in plasma, and that they would have lower binding to plasma proteins. The free fraction has previously been studied in human plasma with *in vitro* ultrafiltration methods using the pure parent tracer of [^18^F]GE180, and the parent free fraction was 0.035 (0.011) [2].

### Anti-CD4 and anti-CD8 staining

To clarify the the result indicated in the manuscript Fig 5. **a** and **b,** where significant differences were detected between the CD4 and CD8 OD of the control animals (i.e. non-treated) at the focal lesion core compared to the perilesional area (*p* < 0.001) (Suppl. Fig. 2).The results are presented as mean (SD).

**Suppl. Figure 2.** Optical density (OD) of the control animals (i.e. non-treated) stained with anti-CD4 and anti-CD8. The lesion core OD was significantly higher in the lesion core when compared to the perilesional area (*p* < 0.001). A similar difference was not detected in the DMF treated rats.

### PET imaging

Additional time-activity data from the time point week 0 and week 1 are provided in Suppl. Fig.3 **a** and **b**.

**a**

**b**

**Suppl. Figure 3.** Average time-activity curves obtained from DMF treated (solid line) and control (dashed line) animals (treated n = 4, controls n=4) at week 0 (**a**) and week 1 (**b**). Imaging was obtained as static imaging with 5 frames between 25-50 minutes post injection. SUV values are indicated as mean (SD).

## References

1. Boutin H, Murray K, Pradillo J, Maroy R, Smigova A, Gerhard A, et al. 18F-GE-180: a novel TSPO radiotracer compared to 11C-R-PK11195 in a preclinical model of stroke. European Journal of Nuclear Medicine and Molecular Imaging. 2015;42:503-11. doi:10.1007/s00259-014-2939-8.

2. Zanotti-Fregonara P, Pascual B, Rizzo G, Yu M, Pal N, Beers D, et al. Head-to-Head Comparison of (11)C-PBR28 and (18)F-GE180 for Quantification of the Translocator Protein in the Human Brain. J Nucl Med. 2018;59:1260-6. doi:10.2967/jnumed.117.203109.

3. Liu B, Le KX, Park MA, Wang S, Belanger AP, Dubey S, et al. In Vivo Detection of Age- and Disease-Related Increases in Neuroinflammation by 18F-GE180 TSPO MicroPET Imaging in Wild-Type and Alzheimer's Transgenic Mice. J Neurosci. 2015;35:15716-30. doi:10.1523/jneurosci.0996-15.2015.
